# Private patient rooms and hospital-acquired methicillin-resistant Staphylococcus aureus: A hospital-level analysis of administrative data from the United States

**DOI:** 10.1371/journal.pone.0235754

**Published:** 2020-07-09

**Authors:** Sae-Hwan Park, Erica L. Stockbridge, Thaddeus L. Miller, Liam O’Neill

**Affiliations:** 1 Center for Health Care Innovation, Perelman School of Medicine, University of Pennsylvania, Philadelphia, Pennsylvania, United States of America; 2 Department of Health Behavior & Health Systems, School of Public Health, University of North Texas Health Science Center, Fort Worth, Texas, United States of America; 3 Department of Rehabilitation and Health Services, College of Health and Public Service, University of North Texas, Denton, Texas, United States of America; Johns Hopkins University, UNITED STATES

## Abstract

**Objective:**

To use hospital-level data from the US to determine whether private patient rooms (PPRs) are associated with fewer in hospital-acquired methicillin-resistant Staphylococcus aureus (HA-MRSA) infections.

**Methods:**

We retrospectively analyzed Texas Inpatient Public Use Data with discharges between September 2015 and August 2016 merged with American Hospital Association annual survey data. We used negative binomial regression to estimate the association between the proportion of PPRs within a hospital and the count of discharges with HA-MRSA infections, adjusting for potentially confounding variables.

**Results:**

We analyzed data for 340 hospitals and 2,670,855 discharges. HA-MRSA incidence within these hospitals was 386 per 100,000 discharges (95% CI: 379, 393) and, on average, 62.73% (95% CI: 58.99, 66.46) of rooms in these hospitals were PPRs. PPRs were significantly associated with fewer HA-MRSA infections (unadjusted IRR = 0.973, 95% CI: 0.968, 0.979; adjusted IRR = 0.992, 95% CI: 0.991, 0.994; p<0.001 for both); at the hospital level, as the percentage of PPRs increased, HA-MRSA infection rates decreased. This association was non-linear; in hospitals with few PPRs there was a stronger association between PPRs and HA-MRSA infection rate relative to hospitals with many PPRs.

**Conclusion:**

We identified 0.8% fewer HA-MRSA infections for each 1% increase in PPRs as a proportion of all rooms, suggesting that private rooms provide substantial protection from HA-MRSA. Small changes may not induce significant improvements in HA-MRSA incidence, and hospitals seeking tangible benefits in HAI reduction likely need to markedly increase the proportion of PPRs through large-scale renovations. The effect of private rooms is disproportionate across hospitals. Hospitals with proportionately fewer PPRs stand to gain the most from adding additional PPRs, while those with an already high proportion of PPRs are unlikely to see large benefits. Our findings enable hospital administrators to consider potential patient safety benefits as they make decisions about facility design and renovation.

## Introduction

In the United States (US), one of every 25 hospitalized patients contracts a hospital-acquired infection (HAI) annually [1]. HAIs cause medical complications and increase morbidity, mortality, and healthcare costs [2, 3]. Staphylococcus aureus is one of the costliest and most dangerous HAIs. “Staph infections”–including both methicillin-sensitive Staphylococcus aureus (MSSA) and methicillin-resistant S. aureus (MRSA) infections–can lead to fatal complications such as pneumonia and sepsis. MSSA and MRSA infections can spread locally and globally, colonize in numerous human body parts, and persist in various environments outside of hosts [4]. MRSA is of particular concern; its resistance to many low-cost antibiotics limit treatment options and increase costs. Further, treatment outcomes for MRSA infections are poorer relative to MSSA outcomes [5]. More than 80,000 new hospital-acquired MRSA (HA-MRSA) cases and more than 11,000 HA-MRSA associated deaths are reported annually [6, 7].

The US Centers for Disease Control and Prevention (CDC) encourage HA-MRSA and other multidrug-resistant pathogen control [8, 9]. Recommended methods to do so include hand hygiene, disinfection, environmental cleaning, contact precautions, antimicrobial stewardship, reducing hospital stays, ensuring appropriate staff-to-patient ratios, and staff cohorting [10, 11]. The CDC also explicitly describes the infection-control benefits of private patient rooms (PPRs) in their guidelines [9].

Studies examining the association between PPRs and HA-MRSA have notable limitations, however, and provide little strong evidence to support recommendations for HA-MRSA control through PPRs. For example, most studies examining PPRs and HA-MRSA were conducted outside the US [12–23]; few studies focused on US hospitals [24–26]. This is a concern given international differences in healthcare delivery structures and hospital organizational characteristics [27, 28] and variations in regulations, control policies, and surveillance measures related to MRSA [29–31]. In addition, most studies examined only a few facilities, which not only limits generalizability but may result in bias given the inability to control for organizational or environmental factors (e.g., staffing, physical spaces) [12–20, 23–26]. Further, many studies were focused exclusively on intensive care unit (ICU) patients [12–15, 18, 22–24, 26] even though the broader inpatient population is at risk of HA-MRSA [19, 25, 32]. In addition to these limitations, study results have been inconsistent. Of 15 reviewed, eight found PPRs significantly reduced HA-MRSA, [12, 13, 17, 18, 21–23, 26] six found no significant effects [14–16, 19, 24, 25], and one had mixed results [20].

We aimed to build on previous research on the association between PPRs and HA-MRSA by examining data from a large number of hospitals in the US. This study differs from most previous research on the PPR-MRSA relationship in that the unit of analysis is the hospital. It is likely that the relationship between PPR and HA-MRSA has at least two distinct dimensions: (1) the decreased MRSA risk afforded to individual patients who stay in PPRs (i.e., an internal effect) and (2) hospital-wide reduced cross-transmissions because of PPRs (i.e., an external effect). Given probable collinearity between the external and internal effects there may be inaccuracy in estimating the infection-reduction attributable to PPRs when conducting patient-level analyses. However, hospital-level analysis mitigates these concerns by evaluating the combined internal and external effects. We hypothesized that an increasing proportion of PPRs in a hospital will be significantly associated with fewer HA-MRSA infections.

## Materials and methods

This study was reviewed and approved as exempt category research by the North Texas Regional Institutional Review Board at the University of North Texas Health Science Center.

### Data sources

The Texas Inpatient Public Use Data File (IP-PUDF), which includes deidentified information about inpatient discharges from hospitals in Texas, was our primary data source [33]. These data are collected by the Texas Department of State Health Services (DSHS) and all state-licensed hospitals are required to provide discharge data for inclusion in this file [34, 35]. The IP-PUDFs contain information regarding patient demographics and information about their inpatient care. The latter includes length of stay, discharge status, diagnosis codes (primary, admitting, and up to 24 non-primary diagnoses), surgery procedure codes, total patient charges, and charges for specific services (such as patient room and ICU).

We analyzed IP-PUDF inpatient stay data from hospitals with 25 or more licensed beds and examined discharges occurring between September 1, 2015, and August 31, 2016. To protect patient confidentiality the IP-PUDF does not provide hospital identifiers on discharge data from hospitals with fewer than 50 annual discharges in total or fewer than 5 annual discharges of one gender; we excluded discharge data with no hospital identifiers. We also excluded data from hospitals with no acute inpatient care facilities (e.g., psychiatric, rehabilitation, and long-term care facilities). IP-PUDF data were restructured to create an analytic data set with one record per hospital.

We obtained additional information about hospitals from the American Hospital Association (AHA) annual survey data [36]. These data contain organizational and structural information about more than 6,000 hospitals and more than 450 healthcare systems. We pulled hospital ownership and other hospital characteristics from the AHA data file. These data were then merged with the restructured IP-PUDF. We validated the number of observed HA-MRSA bacteremia events occurring between September 1, 2015, and August 31, 2016, for each hospital using the Centers for Medicare and Medicaid Services’ (CMS) Hospital Compare Data Archive [37].

### Variables

#### Outcome variable

The count of inpatient stays with HA-MRSA infection diagnoses within each hospital was the outcome of interest. Operational definitions of HA-MRSA included MRSA septicemia (ICD-10-CM = A41.02), MRSA pneumonia (ICD-10-CM = J15.212), and other types of MRSA (ICD-10-CM = A49.09 or B95.62) infections that were not present on admission (i.e., present on admission code = 0). MRSA colonization diagnoses were not included, as this study focused only on infections acquired within the hospital setting and it is difficult to accurately determine whether MRSA was colonized within or outside of a hospital [32, 38, 39]. Our operational definition of HA-MRSA was consistent with past research [30].

#### Primary explanatory variable

The percentage of PPRs within each hospital was the primary explanatory variable. PPRs were defined as single-bed patient rooms, in contrast to patient rooms with two or more patient beds. Such non-PPR “bay rooms” include both semi-private and ward rooms. As with prior work [40], the percentage of PPRs in a hospital was calculated by dividing the count of regular private room discharges by the count of regular room (i.e., non-ICU) discharges. Room assignments were identified based on hospital room charges.

#### Explanatory covariates

We included numerous variables in our multivariable model to adjust for potential confounders in the relationship between HA-MRSA and PPRs. Many of these variables were summary statistics describing the hospitals’ patients and/or the services they received. Because of associations between comorbidity burden and HA-MRSA risk the mean Elixhauser comorbidity index score [41] for each hospital’s patients was included in the model [32, 42, 43]. Variables representing the percentage of patients who were black and Hispanic were included because these populations have high MRSA incidence [44–46], and we included a dichotomous variable that indicated whether or not the inpatient stay included a major therapeutic operating room procedure [47, 48] due to the potential for increased infection risk.

We also controlled for hospital characteristics known to be associated with MRSA [49–51], including teaching facility status (teaching or non-teaching facility), ownership (public, non-profit, or for-profit), hospital location (rural or metropolitan) [52], and number of licensed beds. The percentage of uninsured or Medicaid-insured patients were included as a proxy for safety net hospitals [53, 54]. Nurse staffing levels, as defined by patient-to-nurse ratio based on productive nursing hours versus patient days [55], were included [56–58], as were occupancy rates and physical area per bed [40].

All unbounded continuous variables were log-transformed to reduce data variability [59] and increase interpretability of results. By logging an independent variable (e.g., variable X) to base 10, one can interpret the regression coefficient and confidence interval as the change in the dependent variable (Y) per 10-fold increase in X [60]. Additionally, percentage variables (i.e. those ranging from zero to 1) were multiplied by 100 so that unit marginal changes were equal to 1 and ranged from zero to 100.

### Statistical analysis

First, we examined the unadjusted associations between HA-MRSA counts and each explanatory variable using simple negative binomial regression models (NBM) with no covariates. We normalized the HA-MRSA count, our outcome variable, for these unadjusted analyses; specifically, the count for each hospital was divided by the total number of discharges for that hospital, multiplied by 100,000, then rounded to the nearest integer. We then used a multivariable NBM to examine the adjusted association between HA-MRSA counts and PPRs. In order to facilitate interpretation of the results, the simple (non-normalized) count was used as the outcome variable for all multivariable analyses. Using the simple count as an outcome variable was appropriate for the multivariable analyses but not the unadjusted analyses; the multivariable analyses adjusted for hospital size by including bed count as a covariate, while unadjusted analyses (by definition) did not.

NBM was chosen rather than Poisson regression because the dependent variable (HA-MRSA count) was overly dispersed; the variance was 13.6 times larger than the mean, potentially due to the unobserved heterogeneity and clustering of HAI [61, 62]. NBM was confirmed as suitable for our data using a likelihood-ratio test of the inversed overdispersion parameter. We examined variance-inflation factors (VIFs) to test collinearity among our model variables. We then used the results of the multivariable NBM to estimate the average marginal effects of PPR on HA-MRSA.

Next, we explored unadjusted differences in HA-MRSA incidence and other hospital characteristics for 3 different groups containing roughly the same number of hospitals: (1) Group 1 hospitals (*n =* 113) which contained fewer than 62% PPRs among all patient rooms; (2) Group 2 hospitals (*n =* 114) which contained 62%-82% PPRs; and (3) Group 3 hospitals (*n =* 114) wherein more than 82% of patient rooms were PPRs. We evaluated the significance of pairwise differences in hospital characteristics using chi-squared tests for categorical variables and t-tests for continuous variables, and evaluated differences across the three groups using chi-squared tests for categorical variables and one-way ANOVAs for continuous variables. Additionally, we ran three additional multivariable NBMs to examine adjusted associations between HA-MRSA infection counts and PPRs for each of the three hospital groups. We conducted post-hoc analyses stratifying the data by hospital ownership and risk to determine if results are sensitive to differences in these hospital characteristics.

Finally, to validate our HA-MRSA definition, we conducted a Pearson correlation analysis which examined the extent to which our operational definition of hospital-level HA-MRSA aligned with hospitals’ reported MRSA bacteremia events in CMS’s Hospital Compare Data Archive [37]. All statistical tests were two-sided with significance tested at p < 0.05, and we conducted analyses using Stata MP 13.0 [63].

## Results

The IP-PUDF included data for 3,080,382 inpatient hospital discharges occurring between September 2015 and August 2016; 108,182 (3.5%) of these were from deidentified hospitals and excluded from analysis. The remaining 2,972,400 discharges were associated with 618 hospitals. Hospitals without acute inpatient care facilities (n = 216; 35.0%) or fewer than 25 licensed beds (n = 62; 10.0%) were excluded as well, resulting in an analytic data set representing 340 hospitals and 2,670,855 discharges. HA-MRSA incidence across these hospitals averaged 386 per 100,000 discharges (95% Confidence Interval [CI]: 379, 393) and, on average, 62.73% (95% CI: 58.99, 66.46) of rooms in these hospitals were PPRs. See Table 1 for additional descriptive statistics describing these hospitals and the characteristics of their discharged patients.

**Table 1:** Characteristics of 340 US hospitals and unadjusted associations between hospital characteristics and rates of methicillin-resistant Staphylococcus aureus (HA-MRSA) infection for discharges occurring between September 2015 and August 2016.

Upper Bound	Mean or Percentage (95% CI)	Unadjusted Incidence Rate Ratio* (IRR)	95% Confidence Interval of IRR		p-value*
			Lower Bound	Upper Bound	
PPRs (Mean %)	62.73 (58.99, 66.46)	0.973	0.968	0.979	<0.001
Teaching facility (%)	21.87 (16.61, 28.24)	1.353	0.837	2.187	0.208
Rural location (%)	20.94 (15.68, 26.19)	2.919	1.865	4.571	<0.001
Ownership type (%)					
For-Profit	47.44 (40.99, 53.88)	1.000 (ref)			
Public	14.10 (9.61, 18.59)	2.011	1.077	3.755	0.028
Non-Profit	38.46 (32.18, 44.74)	1.687	1.121	2.539	0.012
# Licensed beds (Mean)	236.97 (200.77, 273.16)	0.999	0.998	0.999	0.025
Publicly insured or uninsured (Mean %)	60.67 (58.61, 62.73)	1.013	1.001	1.025	0.028
Hispanic patients (Mean %)	25.52 (22.56, 28.48)	1.001	0.994	1.008	0.627
Black patients (Mean %)	10.36 (9.23, 11.49)	1.019	1.003	1.034	0.013
Physical space per bed in square feet (Mean)	2565 (1933, 3197)	0.634	0.433	0.929	0.019
Mean Elixhauser score (Mean)	2.90 (2.68, 3.13)	1.064	0.969	1.168	0.190
Nurse-to-patient ratio (Mean)	0.964 (0.776, 1.152)	0.976	0.819	1.162	0.789
Major therapeutic procedures (Mean %)	36.05 (33.95, 38.15)	1.001	0.991	1.012	0.728
Occupancy rate (Mean %)	39.33 (37.00, 41.67)	0.978	0.965	0.992	0.002

PPRs, private patient rooms.

* Unadjusted incidence rate ratios and p-values were generated using bivariate negative binomial logistic regression models.

### Associations between PPRs and HA-MRSA

Unadjusted associations between explanatory variables and HA-MRSA rates are shown in Table 1 and adjusted associations are in Table 2. These tables contain the incidence rate ratios and p-values for all explanatory variables. In both unadjusted and adjusted analyses, PPRs were associated with fewer HA-MRSA infections (p<0.001 for each); specifically, as the percentage of PPRs within a hospital increased, HA-MRSA infection rates decreased.

**Table 2:** Adjusted associations between the characteristics of 340 US hospitals and rates of methicillin-resistant Staphylococcus aureus (HA-MRSA) infections for discharges between September 2015 and August 2016.

Variable	Adjusted Incidence Rate Ratio (aIRR)*	95% Confidence Interval of aIRR		p-value*
		Lower Bound	Upper Bound	
PPR (%)	0.992	0.991	0.994	<0.001
Teaching facility	1.257	1.102	1.434	0.001
Rural location (%)	1.072	0.918	1.252	0.380
Ownership type (%)				
For-Profit	1.000 (ref)			
Public	1.235	1.070	1.425	0.004
Non-Profit	1.129	1.021	1.250	0.019
# Licensed beds (log-transformed)	1.616	1.524	1.713	<0.001
Publicly insured or uninsured patients (%)	1.003	1.000	1.006	0.049
Hispanic patients (%)	1.000	0.998	1.002	0.949
Black patients (%)	1.006	1.001	1.010	0.009
Physical space per bed in square feet (log-transformed)	0.941	0.885	1.002	0.056
Mean Elixhauser score	1.034	1.002	1.068	0.038
Nurse-to-patient ratio (log-transformed)	0.536	0.892	0.356	<0.001
Major therapeutic procedures (%)	1.007	1.004	1.011	<0.001
Occupancy rate (%)	1.012	1.009	1.016	<0.001

PPR, private patient room.

* Adjusted incidence rate ratios and p-values were generated using a multivariable negative binomial logistic regression model.

Conversely, the proportion of publicly insured or uninsured patients within a hospital was positively associated with increased HA-MRSA rates in both unadjusted and adjusted analyses (p = 0.028 and p = 0.049, respectively), as was the proportion of black patients (p = 0.013 and p = 0.009, respectively). In contrast, the proportion of Hispanic patients in a hospital was not significantly associated with HA-MRSA in either unadjusted or adjusted analyses (p = 0.627 and p = 0.949). In both unadjusted and adjusted analyses, public (p = 0.028 and p = 0.004, respectively) and non-profit hospitals (p = 0.012 and p = 0.019, respectively) had higher HA-MRSA rates relative to private hospitals.

Tables 1 and 2 give unadjusted and adjusted associations between hospital characteristics and HA-MRSA infection rates. Being a teaching facility, having patients with higher mean Elixhauser comorbidity scores, and having a higher proportion of inpatient stays during which major therapeutic procedures occurred were each significantly associated with increased HA-MRSA infection rates in adjusted analyses.

**Fig 1:**
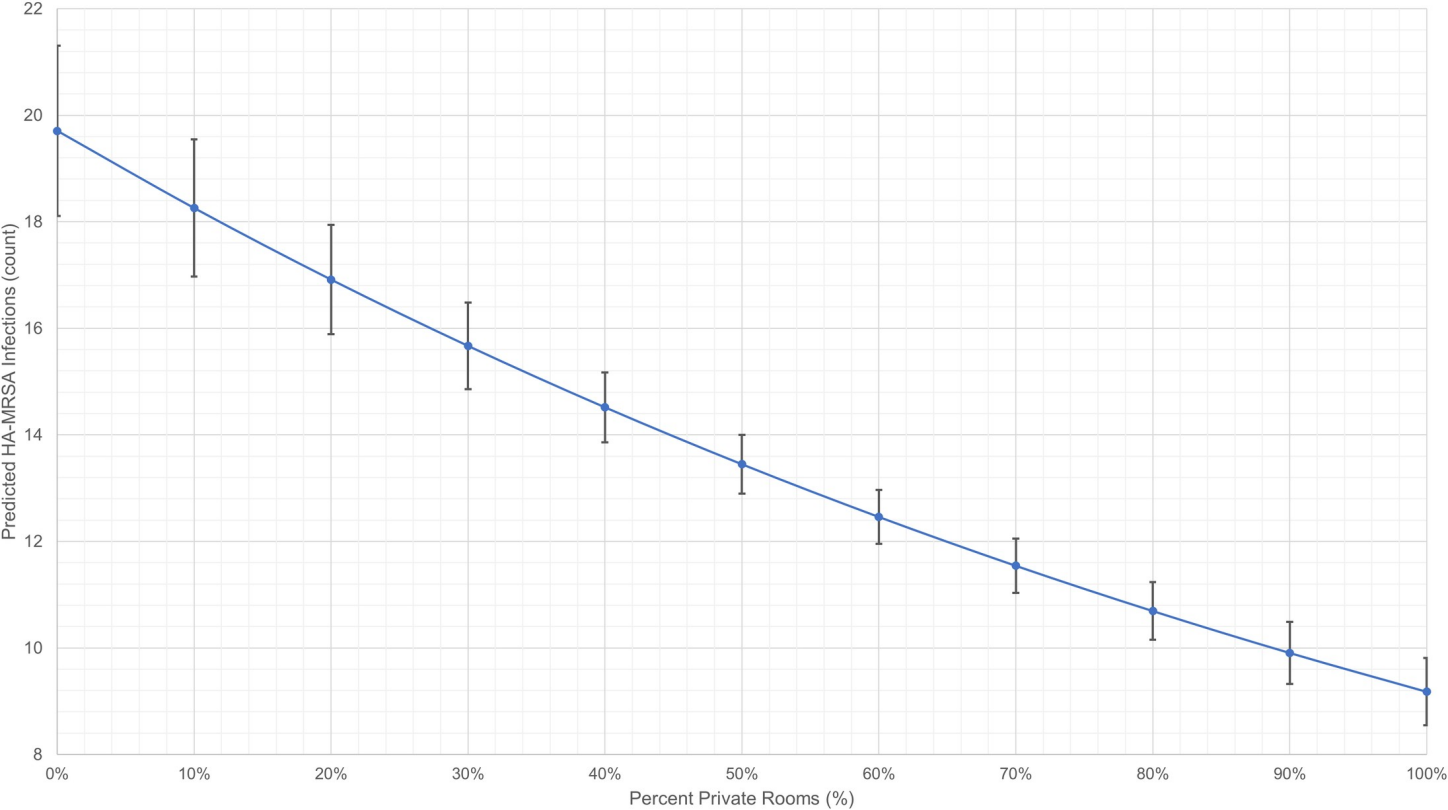
Association between the proportion of PPRs and predicted rates of HA-MRSA. HA-MRSA, hospital-acquired methicillin-resistant Staphylococcus aureus infection. PPRs, private patient rooms. Illustration of the average marginal effects of PPRs on HA-MRSA infection rates, which were estimated using a multivariable negative binomial regression model. Data included discharges between September 2015 and August 2016 from 340 US hospitals.

The average marginal effects of PPRs on HA-MRSA infection rates were estimated from multivariable NBM and illustrated in Fig 1. This graph shows that the relationship between PPRs and HA-MRSA is non-linear, and the confidence intervals illustrate that large differences in the proportion of PPRs in a hospital are significantly associated with HA-MRSA reductions (e.g., PPR = 30% versus PPR = 50%) although small changes are not (e.g., PPR = 10% versus PPR = 20%). Our post hoc analyses indicate that these associations robustly persist when data are stratified by hospital characteristics (S1 Appendix). The adjusted analysis illustrated in Fig 1 also suggests that zero-PPR hospitals, regardless of hospital volume, may achieve 80% of maximum prevention effects by renovating 65% of legacy bay rooms to PPRs.

A likelihood-ratio test of the inversed overdispersion parameter to examine goodness of fit confirmed that NBM was an appropriate statistical test for our data (p<0.001); collinearity tests well below 5 for all variables in the model implied that there was no critical collinearity.

### HA-MRSA and hospitals categorized by PPRs

When we grouped hospitals based on their proportions of private rooms, we found significant unadjusted differences in HA-MRSA incidence. Hospitals with fewer than 62% PPRs had the highest incidence (446 cases/100,000 discharges; 95% CI: 349, 583), hospitals with more than 82% PPRs had the lowest (52 cases/100,000 discharges; 95% CI: 28, 75), and hospitals with 62% to 82% of PPRs fell in between (105 cases/100,000 discharges; 95% CI: 69, 141) (Table 3).

**Table 3:** Characteristics of 340 US hospitals grouped by the proportion of private patient rooms (PPRs), based on discharges between September 2015 and August 2016.

Variable	Group 1 (N = 113; PPR<62%)	Group 2 (N = 114; PPR = 62–82%)	Group 3 (N = 114; PPR>82%)	Overall and pair-wise group comparisons p-values			
	Mean (95% CI)	Mean (95% CI)	Mean (95% CI)	Overall**	1 vs 2*	2 vs 3*	1 vs 3*
HA-MRSA incidence (per 100,000 discharges)	466 (349, 583)	105 (69, 141)	52 (28, 75)	<0.001	<0.001	0.020	<0.001
Teaching facility (%)	25.4 (14.3, 36.4)	21.4 (11.6, 31.3)	18.6 (8.4, 28.9)	0.662	0.589	0.695	0.369
Rural location (%)	31.6 (20.9, 42.3)	17.2 (9.1, 25.3)	12.9 (4.8, 20.9)	0.012	0.032	0.448	0.007
Ownership type (%)							
For-Profit	46.1 (34.6, 57.5)	37.9 (27.5, 48.3)	61.4 (49.7, 73.1)	<0.001	0.013	0.294	0.003
Public	26.3 (16.2, 36.4)	11.5 (4.7, 18.3)	2.85 (0, 6.9)		0.015	0.043	<0.001
Non-Profit	27.6 (17.3, 38.0)	50.6 (39.9, 61.3)	35.7 (24.2, 47.2)		0.009	0.003	0.062
# Licensed beds (Mean)	262 (181, 343)	259 (213, 306)	185 (124, 247)	0.175	0.479	0.027	0.071
Not privately insured (Mean %)	63.9 (60.1, 67.6)	63.1 (60.3, 65.8)	55.3 (51.2, 59.3)	0.001	0.366	0.001	0.001
Hispanic patients (Mean %)	34.1 (28.2, 40.1)	27.4 (22.1, 32.6)	15.6 (12.1, 19.1)	<0.001	0.046	<0.001	<0.001
Black patients (Mean %)	9.2 (7.3, 11.1)	11.4 (9.5, 13.3)	10.6 (8.5, 12.7)	0.298	0.055	0.281	0.175
Physical space per bed (Mean; in square feet)	2,045 (1,549, 2,541)	2147 (1,970, 2,325)	3663 (1,606, 5,720)	0.084	0.343	0.052	0.057
LOS in days (Mean)	4.9 (4.4, 5.4)	4.9 (4.5, 5.4)	4.7 (4.4, 5.0)	0.814	0.976	0.520	0.582
Elixhauser score (Mean)	2.6 (2.2, 3.0)	3.4 (3.1, 3.6)	2.7 (2.2, 3.2)	0.018	0.001	0.002	0.355
Nurse-to-patient (Mean)	0.9 (0.8, 1.1)	0.7 (0.6, 0.8)	1.6 (1.2, 2.0)	<0.001	<0.001	<0.001	0.001
Major therapeutic procedures (Mean %)	33.2 (29.5, 36.8)	28.8 (27.5, 30.1)	46.2 (41.7, 50.8)	<0.001	0.013	<0.001	<0.001
Occupancy rate (Mean %)	34.1 (30.3, 37.9)	45.4 (41.9, 48.9)	37.6 (32.9, 42.4)	<0.001	<0.001	0.004	0.122

HA-MRSA, hospital-acquired methicillin-resistant Staphylococcus aureus infection

PPRs, private patient rooms.

* Unadjusted pairwise differences in the characteristics of the three groups were evaluated using chi-squared tests for categorical variables and t-tests for continuous variables.

** The significance of overall differences across the three groups was evaluated using chi-squared tests for categorical variables and one-way ANOVAs for continuous variables.

The non-linear relationship between the proportion of PPRs and HA-MRSA infection rate is evident in adjusted analyses of hospital groupings (Table 4). In hospitals with fewer than 62% PPRs (Group 1), there is a significant association between PPRs and HA-MRSA infection rate (p = 0.001) with increasing proportions of PPRs associated with a reduction in HA-MRSA infection rates. However, this association is non-significant for hospitals with more than 82% PPRs (Group 3; p = 0.485). The association approaches significance but is not significant for hospitals with between 62%-82% PPRs (Group 2; p = 0.051).

**Table 4:** Adjusted associations between the characteristics of 340 US hospitals and rates of methicillin-resistant Staphylococcus aureus (HA-MRSA) infection, by hospital group, based on discharges between September 2015 and August 2016.

Variable	Group 1*, n = 113 (Fewer than 62% PPRs)				Group 2*, n = 114 (PPR between 62%-82%)				Group 3*, n = 114 (More than 82% PPRs)			
	aIRR**	p-value**	95% CI of aIRR		aIRR**	p-value**	95% CI of aIRR		aIRR**	p-value**	95% CI of aIRR	
			Lower	Upper			Lower	Upper			Lower	Upper
PPR (%)	0.993	0.001	0.989	0.997	0.986	0.051	0.972	1.000	1.007	0.485	0.988	1.027
Teaching facility	1.001	0.993	0.794	1.262	1.541	0.001	1.203	1.974	0.862	0.365	0.626	1.188
Rural location	1.021	0.871	0.796	1.309	1.085	0.555	0.827	1.424	1.140	0.514	0.769	1.690
Ownership type												
For-Profit	1.000 (ref)				1.000 (ref)				1.000 (ref)			
Public	1.117	0.333	0.893	1.397	1.302	0.082	0.967	1.751	2.707	0.000	1.764	4.155
Non-Profit	1.113	0.213	0.940	1.317	1.220	0.024	1.027	1.449	1.291	0.054	0.996	1.674
# Licensed beds (log-transformed)	1.715	<0.001	1.560	1.885	1.558	<0.001	1.393	1.744	1.743	<0.001	1.514	2.007
Publicly insured or uninsured patients (%)	1.002	0.338	0.998	1.007	1.005	0.052	1.000	1.011	1.004	0.251	0.997	1.010
Hispanic patients (%)	1.001	0.599	0.998	1.003	1.000	0.908	0.997	1.003	0.998	0.537	0.993	1.004
Black patients (%)	1.006	0.112	0.999	1.013	1.005	0.246	0.997	1.013	1.010	0.070	0.999	1.021
Physical space per bed in square feet (log-transformed)	0.984	0.766	0.888	1.091	0.950	0.627	0.773	1.168	0.867	0.012	0.775	0.969
Mean Elixhauser score	1.076	0.003	1.024	1.129	1.053	0.091	0.992	1.117	0.867	0.001	0.799	0.941
Nurse-to-patient ratio (log-transformed)	0.406	0.001	0.239	0.691	0.692	0.221	0.383	1.249	0.628	0.045	0.399	0.990
Major therapeutic procedures (%)	1.007	0.003	1.002	1.012	1.009	0.118	0.998	1.021	1.002	0.676	0.994	1.010
Occupancy rate (%)	1.007	0.041	1.000	1.014	1.013	<0.001	1.008	1.018	1.016	<0.001	1.008	1.024

HA-MRSA, hospital-acquired methicillin-resistant Staphylococcus aureus infection

PPR, private patient room.

* Hospitals were grouped based on the proportion of private patient rooms in the facility.

** Incidence rate ratios and p-values were generated using three multivariable negative binomial logistic regression models.

### Validation of HA-MRSA definition

Our operational definition of HA-MRSA based on Texas inpatient discharge data was significantly correlated with hospitals’ reported MRSA bacteremia events in CMS’s Hospital Compare Data Archive (r = 0.78; *p*<0.001). We would not expect perfect concordance given that our definition covers more HA-MRSA conditions than CMS data, which counts only HA-MRSA bloodstream infections [7, 37].

## Discussion

Our results suggest that patient private rooms likely provide substantial protection from HA-MRSA. Each 1% increase of PPRs as a proportion of all rooms is associated with an estimated average 0.8% decrease in HA-MRSA infections (given IRR = 0.992; Table 2). Importantly, these effects are not linear, so a 10% increase of private rooms (i.e., 10 times 1%) may not yield an 8% reduction in HA-MRSA (i.e., 10 times 0.8%). Further, small changes in the proportion of PPRs in a given hospital may not induce significant improvements in HA-MRSA incidence (e.g., PPR = 10% versus PPR = 20%). At the same time, large changes in PPR proportions are significantly associated with fewer HA-MRSA infections (e.g., PPR = 30% versus PPR = 50%; Fig 1), suggesting that hospitals will likely need to markedly increase their proportion of PPRs if they wish to obtain tangible benefits in HAI reduction.

Similarly, the protective effect of private rooms is disproportionate across hospitals and as a hospital approaches “saturation” relative to its proportion of PPRs the effect diminishes and is no longer significant (Table 4). This diminishing marginal rate of improvement can be seen in Fig 1‘s plot of predictive margins. The marginal change in HA-MRSA depends on how many private rooms currently exist in a hospital; hospitals which already have a high proportion of PPRs will benefit little by adding additional PPRs, whereas greater benefit will be returned to hospitals with proportionately fewer PPRs. The non-linear and diminishing marginal effect of PPRs may be explained by the positive externalities that are likely associated with such rooms. All patients who are treated a hospital built with a higher proportion of private rooms may benefit from the safer environment, both directly and indirectly [64, 65].

This idea is analogous to “herd-immunity;” that is, the protective effects of PPRs in a hospital may play a similar role to that of vaccines in a society. HA-MRSA infections are infectious diseases that can be transmitted through doctors and nurses, and thus PPRs are likely linked to HA-MRSA prevention through reductions in cross-transmission. More PPRs in a hospital equates to greater personal space and less crowding, which is associated with better hand hygiene in hospital staff [64, 65]. Communication and coordination among staff are also strongly encouraged in PPRs [66]. Further, hospitals with fewer PPRs typically hire fewer nurses per bed, which may result in understaffing or excessive workload levels that can worsen the risk of HA-MRSA [67, 68]. Additionally, there is better control of the aerial dispersion of pathogens in PPRs relative to multi-bed rooms [69]. Together, these controls suggest that the HA-MRSA bacteria present within a hospital would be unlikely to be transmitted if the hospital has a high proportion of private rooms, much like a vaccine-preventable infectious disease would be unlikely to be transmitted in a highly vaccinated society. Consequently, even patients in multi-bed rooms may be protected against MA-MRSA by receiving treatment in a hospital with a high proportion of PPRs (i.e., the external effect of PPRs).

While PPRs have become a standard design feature of newer hospitals, some legacy hospitals (particularly rural, public, and/or safety-net hospitals) still contain many bay and semi-private rooms [70–73]. Previous research has found rates of MRSA infection to be higher in hospitals with more frequent antibiotic uses that are often associated with public hospitals [3, 6], rural locations [6], and teaching facilities [3–4,18]. Our results are consistent with these previous findings. The reasons for the differences in rates of MRSA infections between rural and urban hospitals are complex and beyond the scope of the present study, although the associations that we identified provide opportunities for future research in this area. For example, as shown in Table 3, Group 1 hospitals were more likely to be publicly owned, a teaching facility, and located in a rural county. They also treated the highest percentage (34%) of Hispanic patients. Patient severity, as measured by the average number of chronic comorbidities, was not significantly higher for Group 1 hospitals. Group 3 hospitals were more likely to be for-profit and located in a metropolitan area.

Our findings suggest that patients who use such hospitals likely face greater risks from HA-MRSA, but also that plausible safety, quality, and economic incentives exist for hospitals to proactively increase their proportion of PPRs. While it is worth noting that the physical design of rural facilities and the relative scarcity of private rooms may be a contributing factor to higher rates of MRSA infections, from the patient's perspective the marginal benefit of being assigned to a private rooms appears to be greatest at those facilities with the lowest percentage of private rooms. At facilities with a high percentage of private rooms (>82%), the marginal benefit of being assigned to a private room was not statistically significant.

Over time, the rise of the private hospital room has coincided with many other changes in hospital design, such as decentralized nursing stations, acuity-adaptable rooms [42], use of antimicrobial surfaces, and improved air filtration [4, 30]. Many experts believe that these design changes that are associated with modern facilities has created a safer hospital [4, 30, 41]. Private hospital rooms have also been associated with more visible sinks in patient rooms and better hand hygiene [66, 70, 71].

Our methods address many limitations identified from previous studies, allowing the most robust and valid analysis of these issues available to date. Many prior studies are hindered by limited generalizability, constrained settings, and/or non-representative samples, as well as inconsistent findings due in part to patient-level analyses which introduce collinearity between decreased MRSA risk afforded to individual patients who stay in PPRs (i.e., the internal effect) and hospital-wide reduced cross-transmissions because of PPRs (i.e., the external effect) [12–26]. We used hospital-level data from hundreds of hospitals with a variety of patient, structural, and organizational characteristics to identify an association between decreased HA-MRSA risk and PPRs. This method mitigates collinearity concerns by examining total combined internal and external effects. Our findings align with those of the one other study that we identified which used hospital-level data to examine this issue, and it improves on that study by examining a larger number of hospitals (340 versus 176) and using data that hospitals were required to report by statute instead of survey data subject to hospital non-response [21]. Further, our work examined HA-MRSA in US hospitals, so our findings are relevant to hospital administrators/owners and policymakers in the US.

From a policy perspective, our results suggest at least two opportunities for improving public reporting and surveillance. First, public disclosure by hospitals of the proportion of PPRs they contain may facilitate HA-MRSA and potentially other nosocomial infection prevention. Such information is not currently easily accessible. We were able to estimate this information based on room charges, but doing so required a significant amount of data processing. Given our finding that the proportion of PPRs is associated with fewer HA-MRSA infections, this lack of public information on PPRs is unfortunate. Were hospitals to publicly report PPR information, patients could incorporate this information into their decision-making processes as they choose hospital providers. Market forces might work to encourage hospitals with proportionately few PPRs to renovate, which could ultimately result in improved patient safety within these hospitals.

Second, MRSA can be spread from patient to patient even when the infection is not in the bloodstream [74]. With that in mind, policymakers might consider expanding MRSA surveillance measures so they cover a more comprehensive set of infections, and our broad measure of HA-MRSA could be used when doing so. We found a strong correlation between current surveillance measures based only on bloodstream infections and our broad measure of HA-MRSA which included MRSA septicemia, MRSA pneumonia, and other types of MRSA infection. Expanding the MRSA definition would provide more information about infection risk without a marked loss in surveillance consistency, as the distinction between better performing hospitals and worse performing hospitals would not change dramatically if MRSA definitions were to be expanded.

While our study provides actionable information about the association between HA-MRSA and PPRs, it has limitations that must be considered when interpreting the results. Due to the lack of available information about PPRs, we estimated PPR percentages for each hospital based on charge data. This estimate was likely affected by room utilization and/or occupancy. While this approach is beneficial in that it only counts rooms that were actually assigned, future research using actual rather than estimated PPR proportions is warranted. Additionally, although our data represents a very large number of hospitals, it comes from only a single US state–Texas. Given Texas’ size as well as diverse population and geography it is likely that our findings are robust, but there is a need to replicate this study to confirm the generalizability of our findings across the US.

The cross-sectional nature of this study has inherent limits and our predicted improvement is better understood as a comparative result (i.e., hospital A versus hospital B) rather than a strong estimate of expected improvement within a given hospital. Time-series analyses would be required to verify that a given hospital can expect a particular degree of improvement. Further, we are unable to make strong causal statements based on this cross-sectional data.

Moreover, both private room effects and hospital-acquired infections involve complicated interactions among staff, patients, and facilities. It is likely that the effects of all possible predictors and confounders are not fully controlled in our analyses. For example, we do not consider length of stay in this analysis. A longer stay may increase a patient’s risk of HA-MRSA infection; however, a patient who contracts HA-MRSA is likely to be required to stay in the hospital longer. As we could not determine causal directionality, length of stay was not included as a covariate. This exclusion aligns with the methodology of the majority of studies on PPRs and HA-MRSA [13–15, 17–21, 23–25]. Other hidden variables may also be at play.

Finally, we have examined only one aspect of private rooms–their potential in preventing HA-MRSA. However, there are many other potential benefits of private rooms, including patient privacy, reduced errors, and increased nurse and patient satisfaction [75–78]. Hospital administrators should not overlook these factors when considering hospital renovations that would increase the proportion of PPRs. Future research that includes a more comprehensive assessment of the patient safety and satisfaction effects of PPRs would be beneficial to such decision-makers.

## Conclusion

We identified significant associations between decreased HA-MRSA risk and PPRs using hospital-level data from hundreds of US hospitals with a variety of patient, structural, and organizational characteristics. Our results suggest that private rooms likely provide substantial protection from HA-MRSA infections; there is an average 0.8% decrease in HA-MRSA incidence for each 1% increase in PPRs as a proportion of all rooms. However, small changes in the proportion of PPRs in a given hospital may not induce significant improvements in HA-MRSA incidence, so hospitals will likely need to markedly increase the proportion of PPRs through large-scale construction or renovation projects if they wish to obtain tangible benefits in HAI reduction. Further, the protective effect of private rooms is disproportionate across hospitals. Hospitals which already have a high proportion of PPRs will benefit little by adding additional PPRs, whereas greater infection control benefits will be returned to hospitals with proportionately fewer PPRs. This actionable information about the association between HA-MRSA and PPRs will enable hospital administrators to consider potential patient safety benefits as they make decisions about facility design and renovation.

## Supporting information

S1 Appendix (PDF)
