# Supplementary material for: Private patient rooms and hospital-acquired methicillin-resistant Staphylococcus aureus: A hospital-level analysis of administrative data from the United States
Source: PLoS One. 2020 Jul 9;15(7):e0235754. doi: 10.1371/journal.pone.0235754 (PMC7347222; doi:10.1371/journal.pone.0235754)

Appendix: Sub-analysis within similar hospitals

1. Hospitals by Ownership Style (Left public hospitals; Center non-profit hospitals; Right for-profit hospitals)

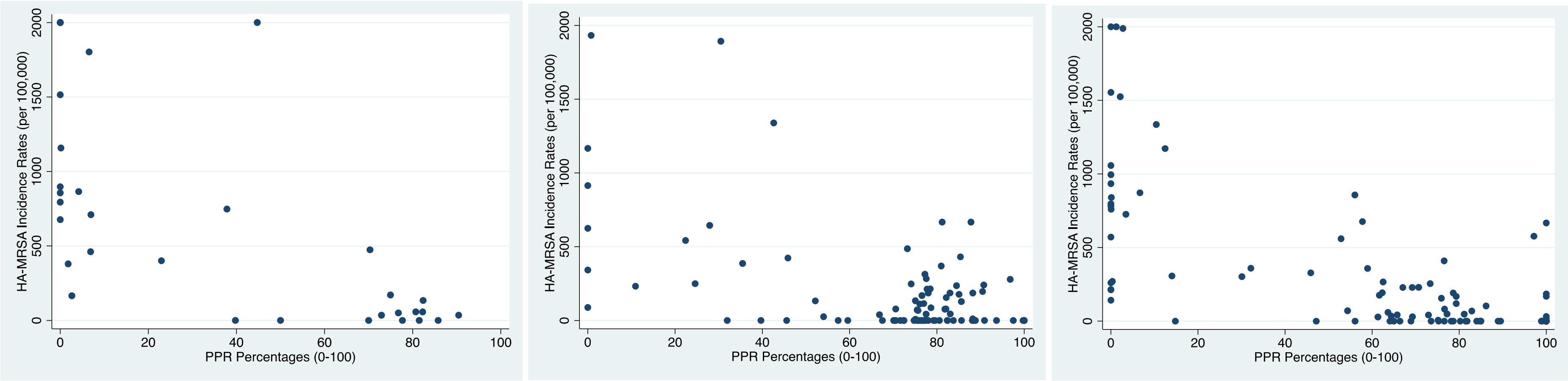

2. High-risk Hospitals (left major therapeutic procedures > 75-th percentile; center teaching facilities only; right rural hospitals only)

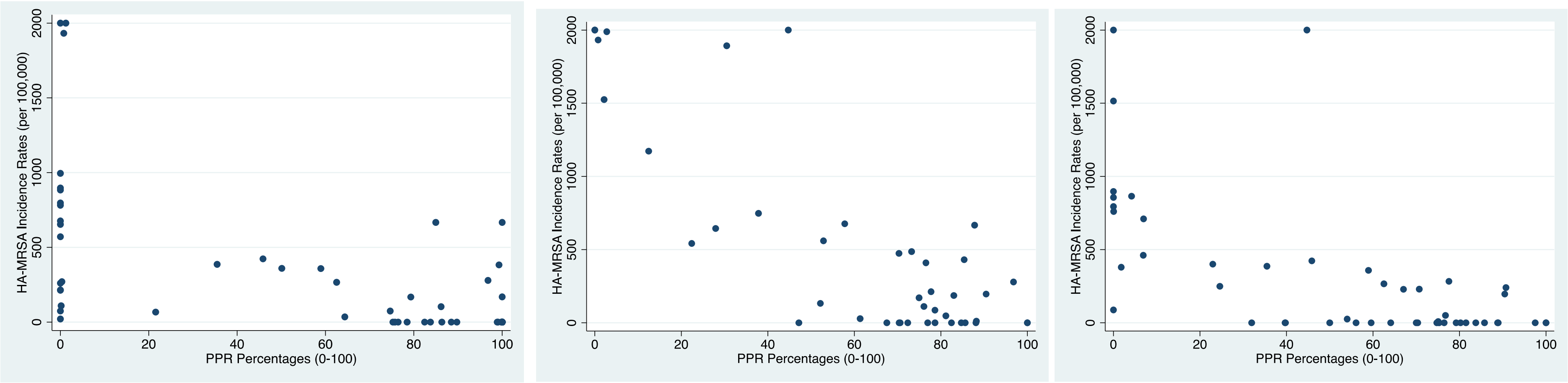

Supplement: S1 Appendix — (PDF) [file pone.0235754.s001.pdf]
